# Supplementary figures and images for: Vibrio cholerae RbmB is an α-1,4-polysaccharide lyase with biofilm-disrupting activity against Vibrio polysaccharide (VPS)
Source: PLoS Pathog. 2024 Dec 2;20(12):e1012750. doi: 10.1371/journal.ppat.1012750 (PMC11637428; doi:10.1371/journal.ppat.1012750)

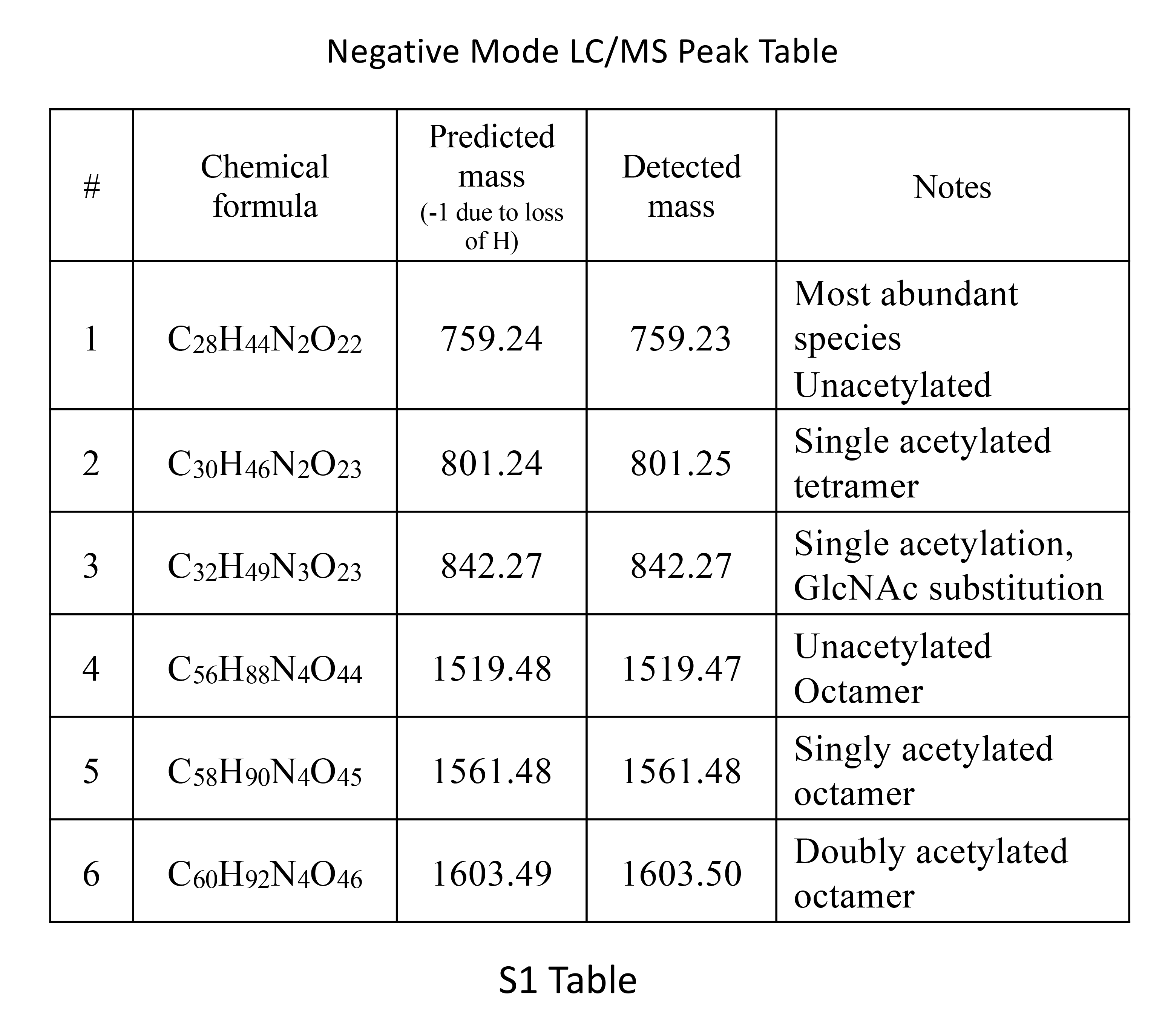

Supplement: S1 Table — (TIF) [file ppat.1012750.s001.tif]

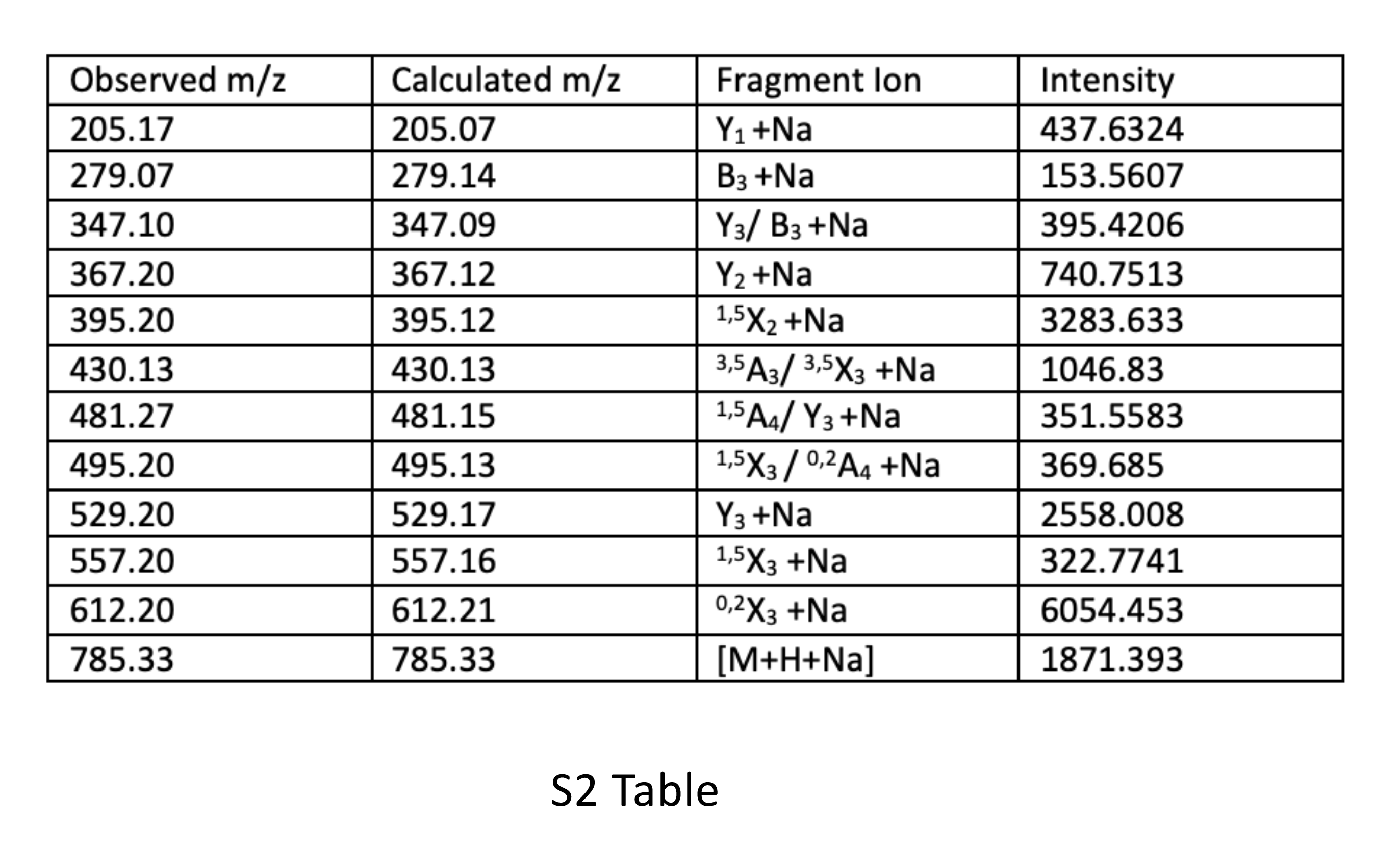

Supplement: S2 Table — Fragmentation annotations follow the Domon-Costello nomenclature [50]. (TIF) [file ppat.1012750.s002.tif]

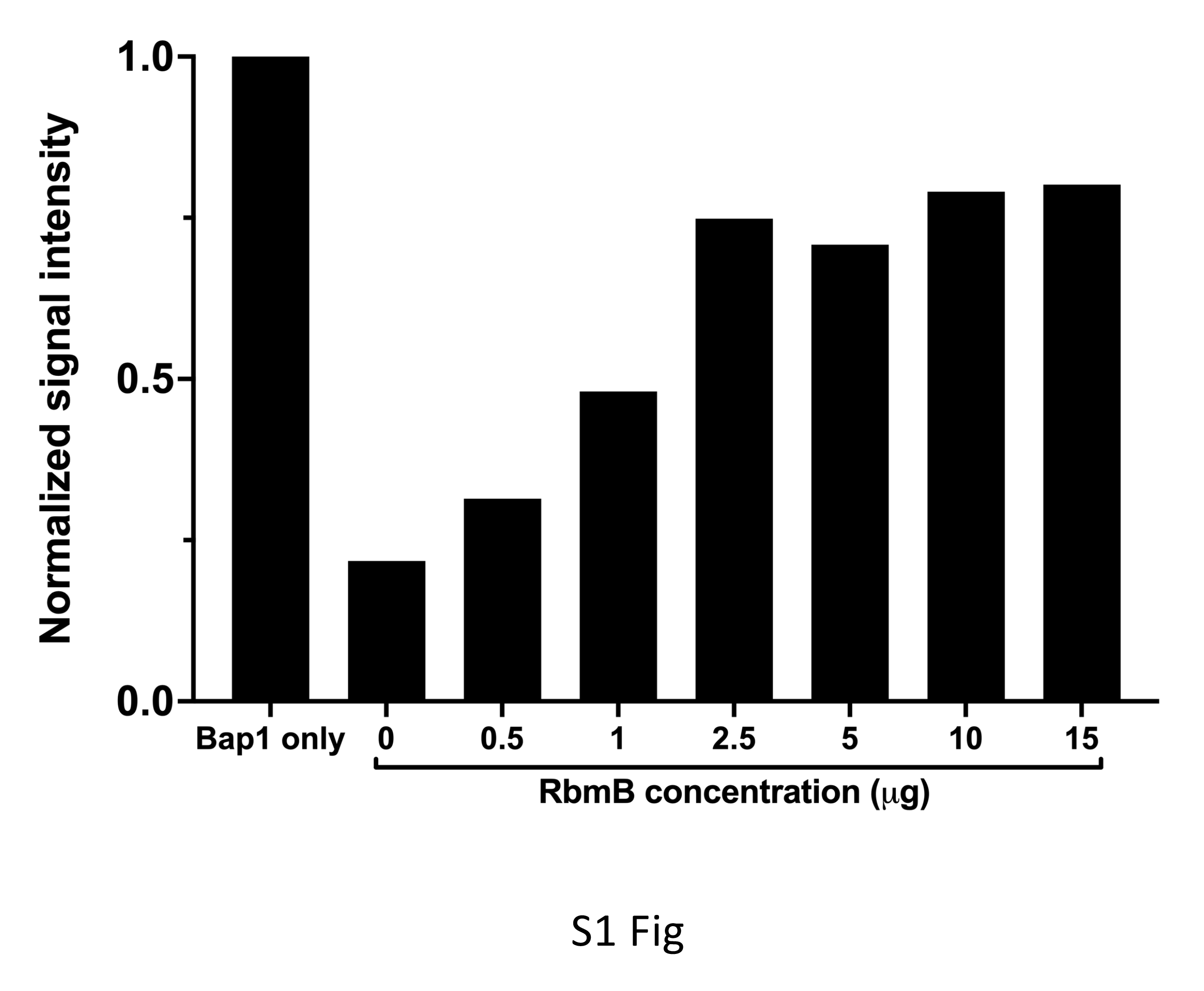

Supplement: S1 Fig — An increase in the amount of free Bap1 is observed, consistent with digestion of the VPS polymer into smaller fragments. Data represent a single replicate. (TIF) [file ppat.1012750.s003.tif]

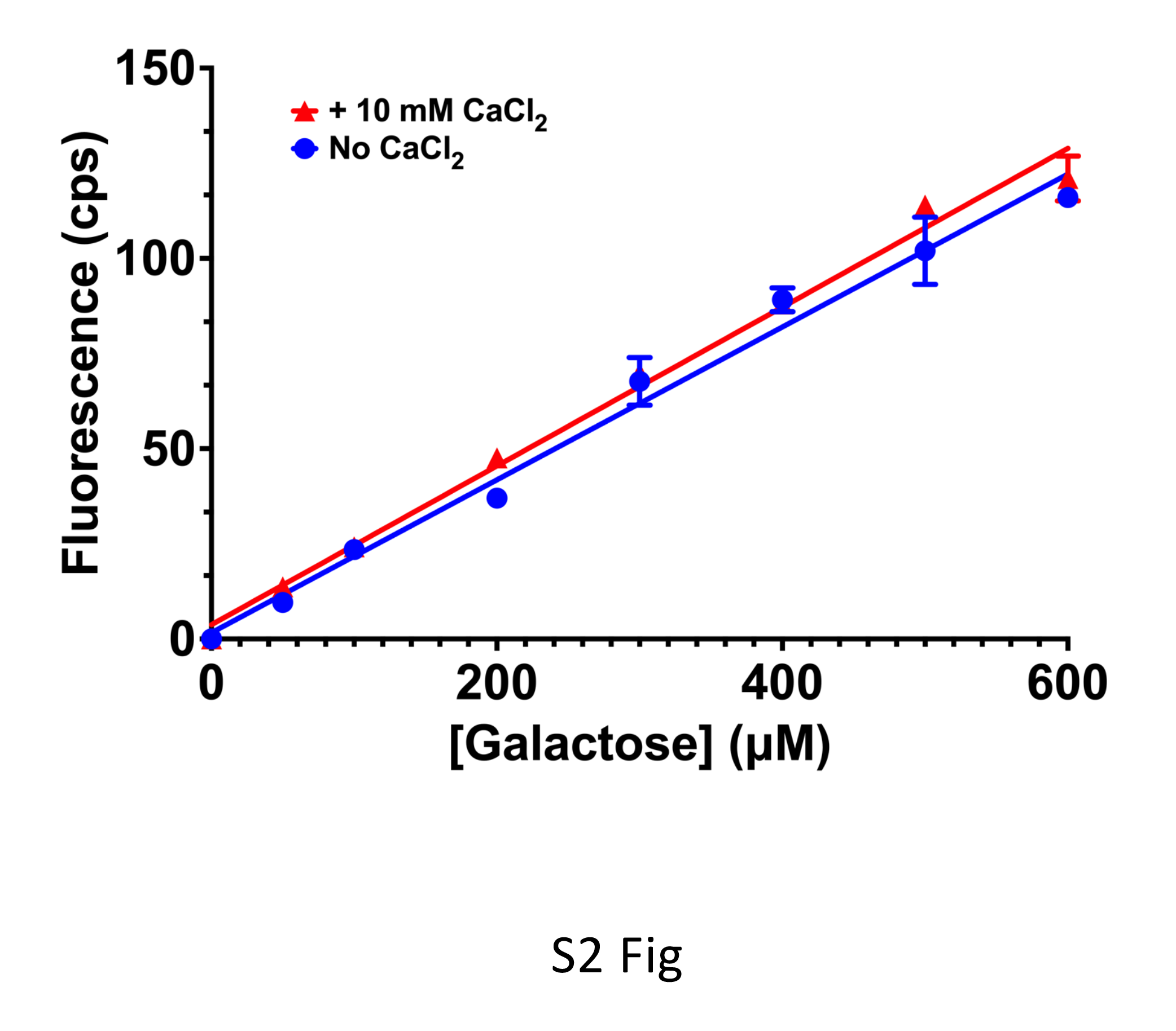

Supplement: S2 Fig — All data are represented as the mean ± SD (n = 3). (TIF) [file ppat.1012750.s004.tif]

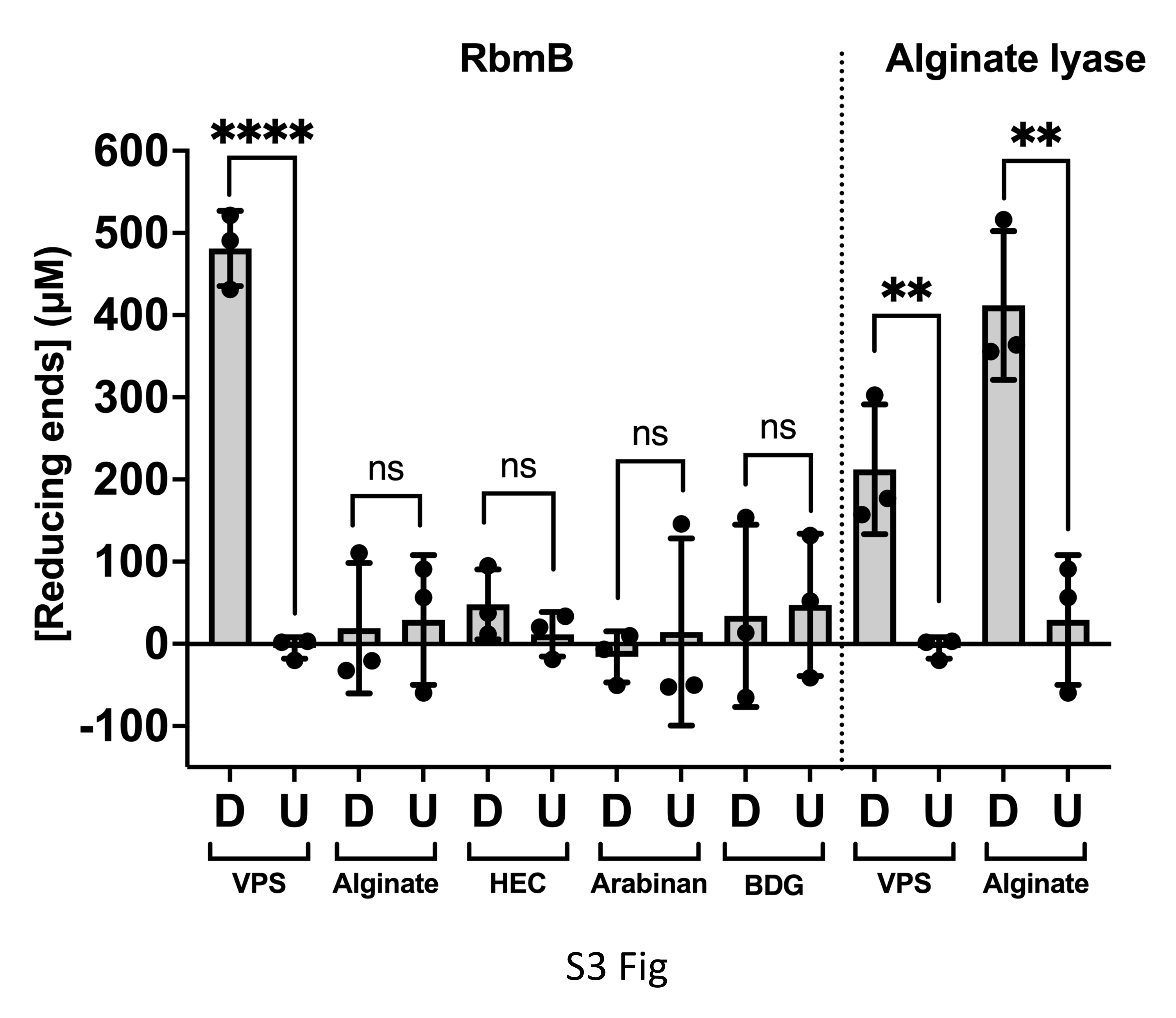

Supplement: S3 Fig — Fluorescence-based quantification of reducing ends before (U) and after (D) digestion is shown for several polymers. HEC represents hydroxyethyl cellulose. All data are represented as the mean ± SD (n = 3). Statistical significance was determined using an unpaired, two-tailed t-test with Welch’s correction. ns = not significant, *p<0.05, **p<0.01. Solid lines indicate which samples were compared in the statistical tests. (TIF) [file ppat.1012750.s005.tif]

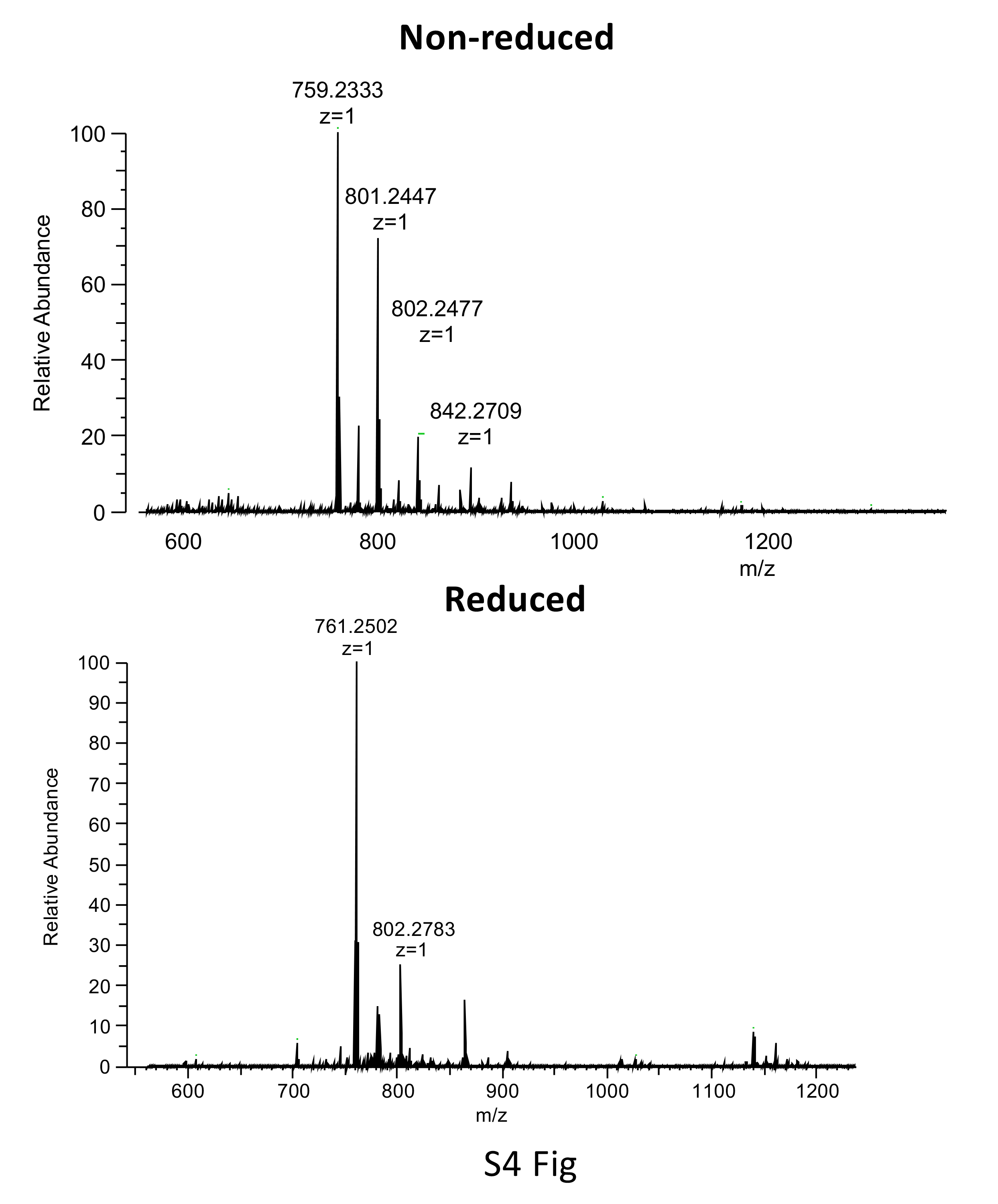

Supplement: S4 Fig — RbmB-digested VPS was incubated with 50 mM KOH, 1M NaBH4 for 3 hours at 60°C. Peaks indicate the expected shift of +2 Da. (TIF) [file ppat.1012750.s006.tif]

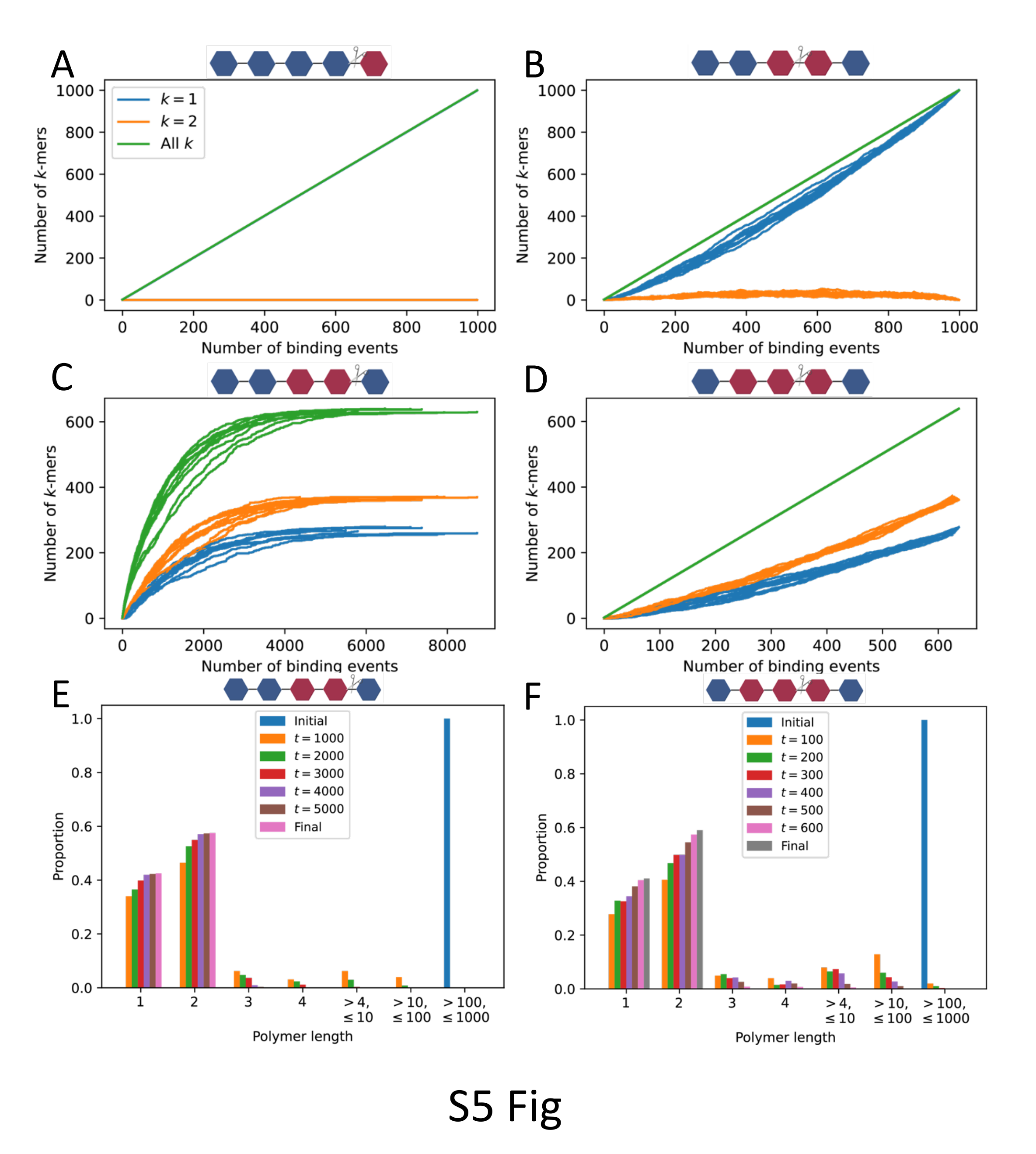

Supplement: S5 Fig — (A) Exoglycosidase strategy with RbmB cutting a single tetrasaccharide from the end of the VPS polymer. k = 1 and k = 2 represent the tetra- and octasaccharide species, respectively. This model predicts a linear accumulation of tetrasaccharide species over binding events. (B) Octasaccharide recognition strategy with cutting in the middle predicts primarily tetrameric species. (C) Octasaccharide recognition strategy with cutting at the end predicts a mixture of tetra- and octasaccharide species. (D) Dodecamer recognition strategy with cutting in the middle also predicts a mixture of tetra- and octasaccharide species. (E) Histogram showing accumulation of k = 1 and k = 2 species over time from a representative simulation in panel (C). (F) Histogram associated with panel (D). (TIF) [file ppat.1012750.s007.tif]

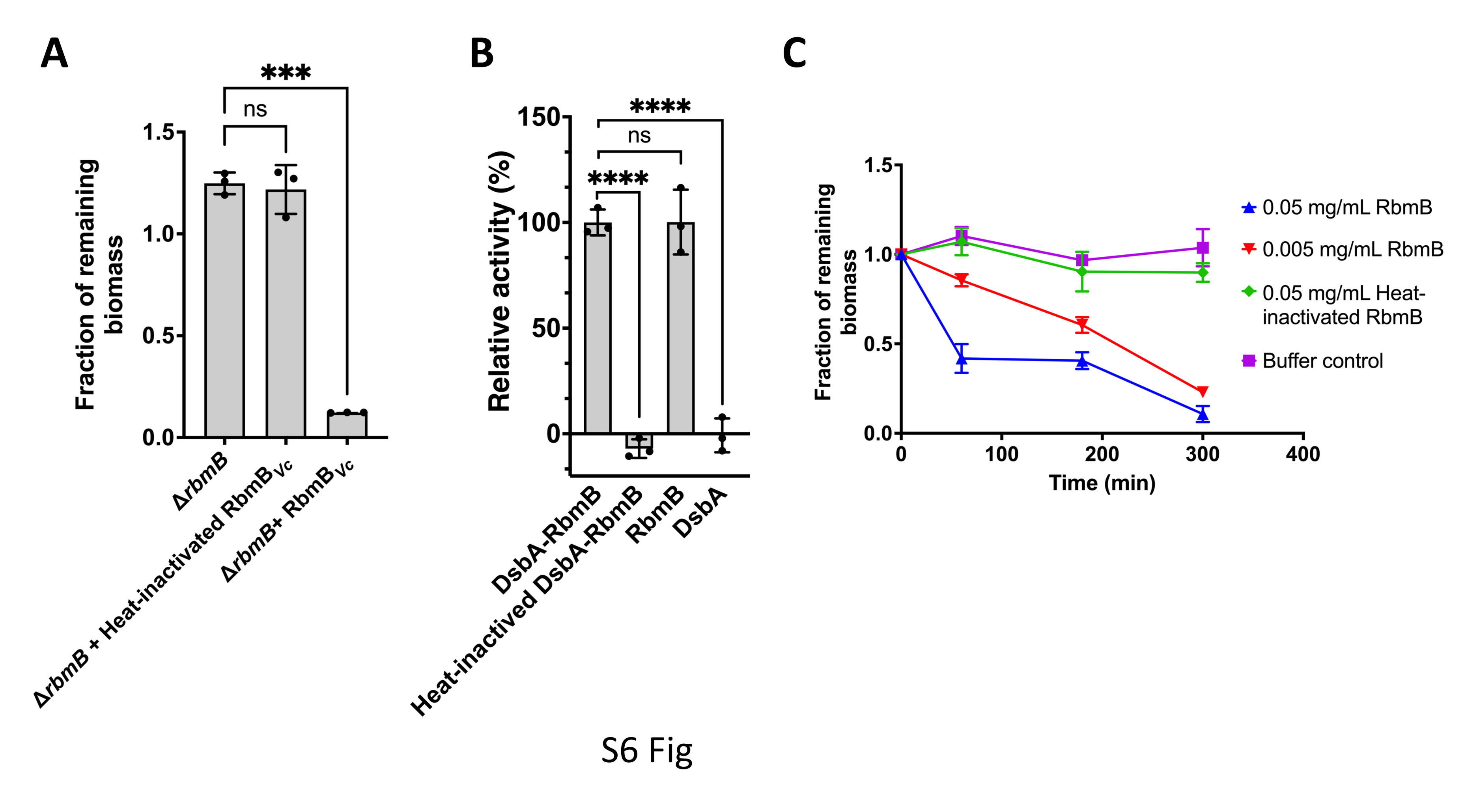

Supplement: S6 Fig — (A) Biomasses of biofilms grown without nutrient limitation before and after introduction of RbmB (at 50 μg/mL) for 1 hour at 30°C were quantified using a confocal microscope. Heat-inactivated RbmB (50 μg/mL) had no significant effect on biofilm disruption. (B) Relative enzymatic activity of 20 μg of recombinant RbmB against 25 μg of purified VPS for 30 min demonstrates a loss of activity following heat-inactivation. Cleavage and removal of the DsbA tag does not alter the RbmB activity and purified DsbA is unable to cleave VPS on its own. (C) Wild-type V. cholerae biofilms grown without nutrient limitation are disrupted in a dose-dependent manner by recombinant RbmB. All data are depicted as mean ± SD. Statistical analyses were performed using an unpaired, two-tailed t-test with Welch’s correction. ns = not significant, ***p < 0.001, ****p < 0.0001. Solid lines indicate which samples are compared for each statistical test. (TIF) [file ppat.1012750.s008.tif]

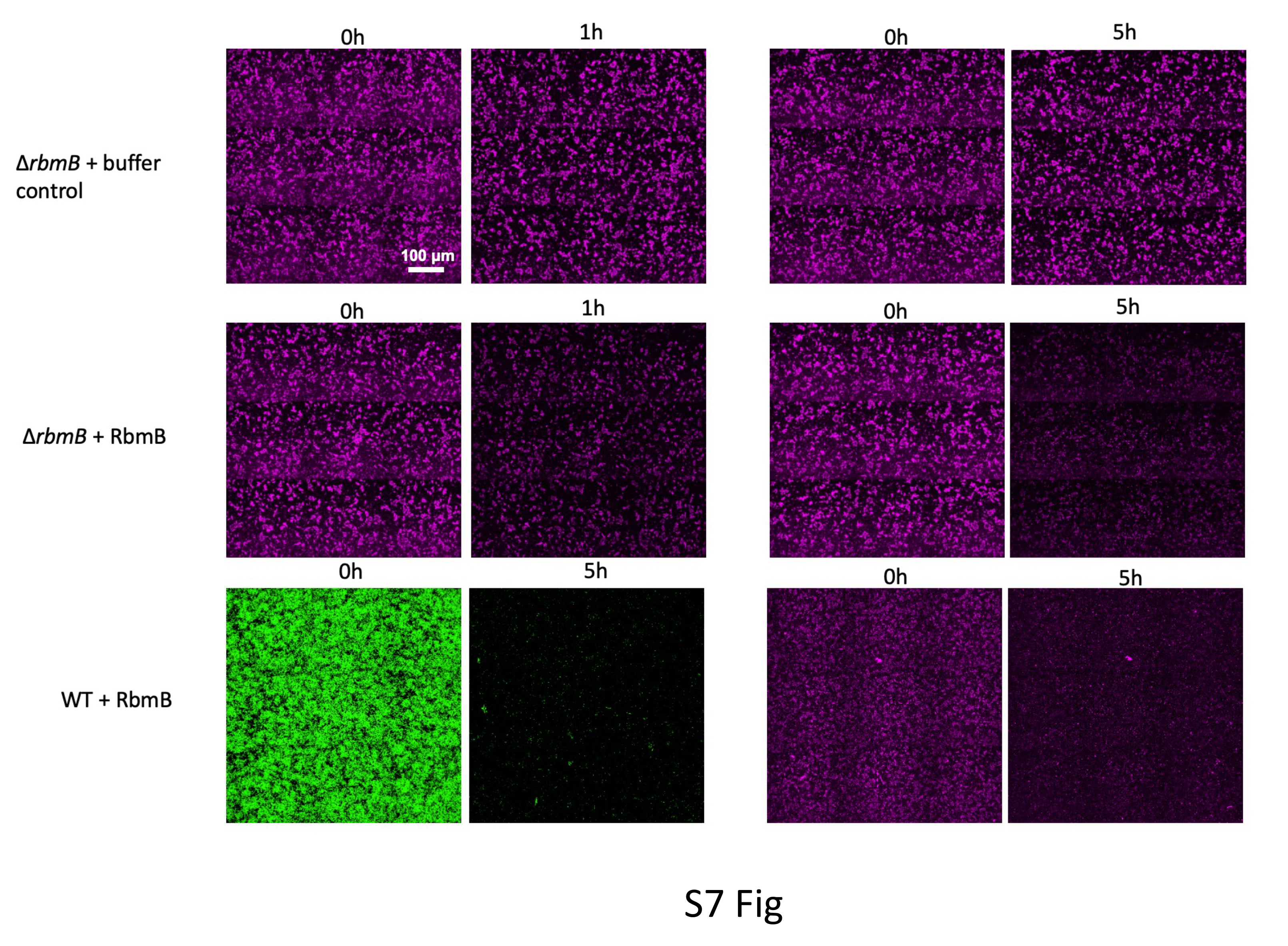

Supplement: S7 Fig — Biofilms were imaged with fluorescently labeled WGA to stain VPS at 0 hours, 1 hour, and 5 hours. The WGA signal remained strong in the ΔrbmB mutant biofilms (top row), while addition of 50 μg/mL RbmB led to a decrease in the WGA signal (middle row). The lower row shows the same experiment with the wild-type strain. The left two panels show cells (mNeonGreen fluorescence) and the right two panels show WGA staining before and 5 hours after addition of RbmB. (TIF) [file ppat.1012750.s009.tif]

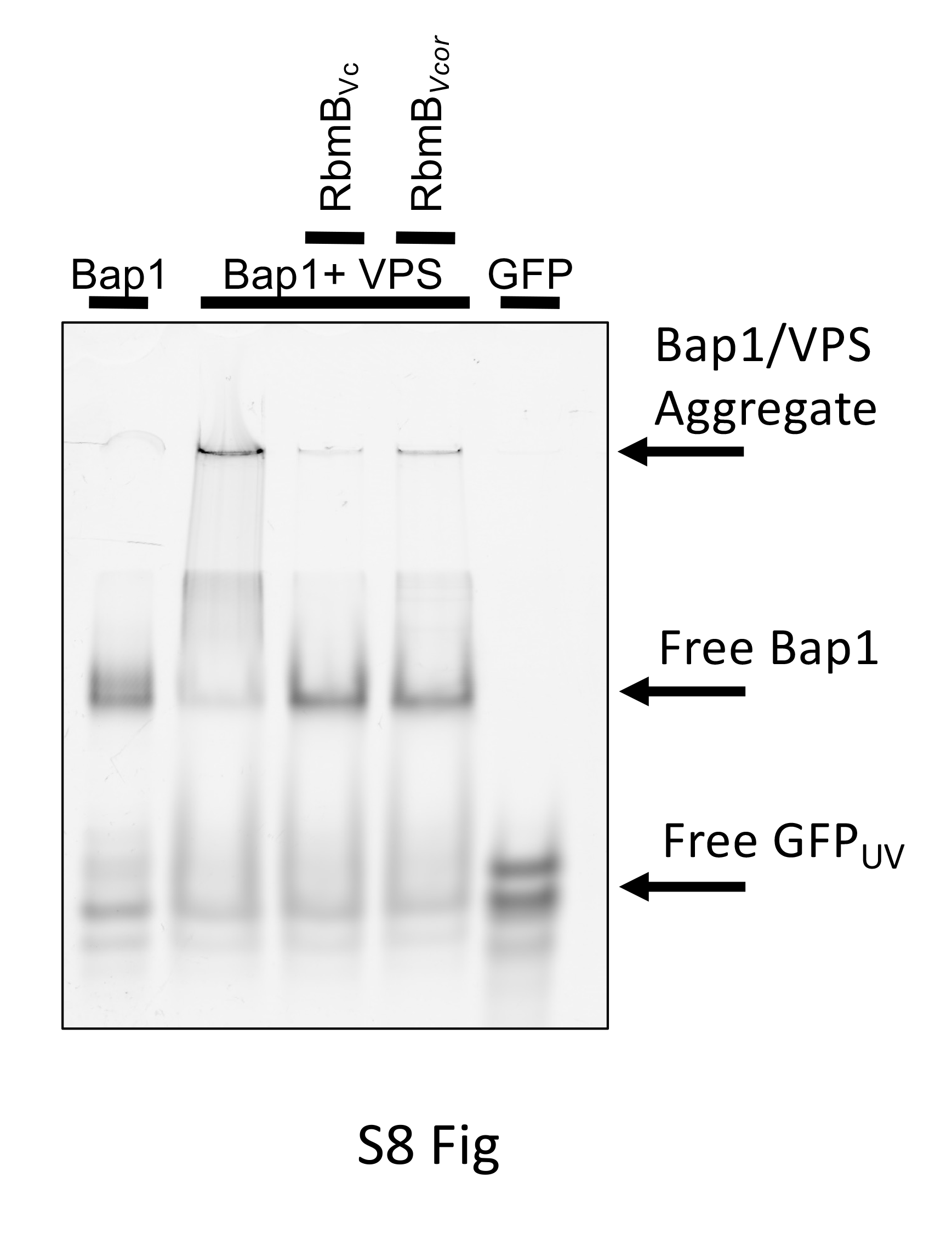

Supplement: S8 Fig — Fluorescence-imaged native-PAGE gel using Bap1-GFPUV fusion to image VPS polymer. Digestion of VPS with RbmB from either species leads to a reduction in Bap1/VPS aggregates. (TIF) [file ppat.1012750.s010.tif]
