# Supplementary material for: Vibrio cholerae RbmB is an α-1,4-polysaccharide lyase with biofilm-disrupting activity against Vibrio polysaccharide (VPS)
Source: PLoS Pathog. 2024 Dec 2;20(12):e1012750. doi: 10.1371/journal.ppat.1012750 (PMC11637428; doi:10.1371/journal.ppat.1012750)
Supplement: Attachment — Submitted filename: Reviewer_Responses.pdf [file ppat.1012750.s011.pdf]

Wesleyan University
Molecular Biology and Biochemistry Department
Molecular Biophysics Program
College of Integrative Sciences

Rich Olson
Associate Professor
224 Hall-Atwater Laboratories
52 Lawn Avenue
Middletown, CT 06459
Phone: 860-685-3070
Fax: 860-685-2141
Email: rolson@wesleyan.edu

September 19, 2024

Dear Editors,

We thank the reviewers for providing such detailed and insightful questions which allowed us to strengthen our manuscript. We appreciate the significant time they and the editors contributed to this process. Please find our responses to the reviewer comments below highlighted in blue.

Sincerely,

Rich Olson, Ph.D.

Jing Yan, Ph.D.

Part II – Major Issues: Key Experiments Required for Acceptance
Please use this section to detail the key new experiments or modifications of existing experiments that should be absolutely required to validate study conclusions.

Generally, there should be no more than 3 such required experiments or major modifications for a "Major Revision" recommendation. If more than 3 experiments are necessary to validate the study conclusions, then you are encouraged to recommend "Reject".

Reviewer #1: (No Response)

Reviewer #2: - Description of experimental conditions in the results section would benefit from more detailed descriptions, e.g. it was not readily apparent what was loaded onto the SDS gel (l. 177-182).

We updated the description in the text and figure legend to better explain the gel that you describe (which is a native gel). We also went through the results and methods sections to add more details about the experiments (including amounts of enzyme and VPS used).

- Figure 3A and Figure S1: There seems to be a discrepancy in the scale of the graph. The author mentioned that a standard curve was made using 0 to 600 mM of galactose (L 522-523), but the scale of Fig 3A and Fig S1 is μM. Could you kindly provide some clarification on this?

This was a font error. It should be μM. We fixed it in the text.

- Figure 4D and Figure S3: #4 and #6 chemical formula, which are presented in Fig S3, are not shown in Fig 4D. Is it because of the small peak?

Yes. We updated the figure to add labels for these peaks for consistency.

- Figure 6A and B: Please clarify the treatment (Y axis). In Figure 6A, does the treatment mean arabinose induction or nutrient limitation for inducing biofilm dispersion? The treatment in Figure 6B may imply the exogenous addition of RbmB or nutrient limitation. The author needs to clearly explain this in the Figure legend.

We apologize for the confusion. By treatment we did mean nutrient limitation for inducing biofilm dispersion. We did so by switching to a medium without carbon source (for details see the Methods section). We have updated y-axis legend and clarified this point in the Figure legend. We had to induce nutrient limitation for the dispersal assay because even if exogenously added RbmB degrades VPS, the biofilm-dwelling cells need to undergo a physiological transition to the planktonic state for proper dispersal.

- Figure 6C: The authors need to check the magnification of each confocal image. It seems that the magnification is different for each image.

The magnification is indeed correct. It may appear to have different magnifications because the biofilm cluster size varies from experiment to experiment. The variability does not affect the results because by taking the ratio of biomass after and before dispersal, each sample is self-calibrated with respect to sample-to-sample variation in biofilm size and mass.

- L219-223, Figure 3E: Fig 3E indicates that RbmB cleavage activity is increased in the presence of calcium. However, at L 220, the author mentioned that the presence of calcium is not required for the activity as treatment with EDTA did not abolish the cleavage. However, EDTA chelates divalent ions other than calcium. The authors should consider using the calcium specific chelator EGTA. Also, regarding the author's explanation, is there any evidence that VPS interacts with calcium?

Thank you for this suggestion. We repeated the assay and used EGTA in addition to EDTA. We saw a similar effect with both and included it in the figure. We also added significance tests to quantify the effect. While some negatively-charged polymers (like alginate) do interact with calcium to form gels, we have not observed changes in VPS solution behavior caused by calcium. Many lyases however, do involve calcium in the cleavage mechanism. We suspect that calcium that calcium acts as a cofactor to facilitate cleavage, but as our results show, it is not essential for activity. We modified the text and added a reference that describes calcium-assisted lyases.

- Figure 3E: The data showed that the baseline has activity. Why does it have cleavage activity?

What we labeled baseline was untreated protein. As this already had been exposed to EDTA, it really was more of a control (and similar to our +EDTA sample). Therefore, we would expect it to act like +EDTA (which still exhibits activity). We removed the baseline sample as it was redundant with +EDTA.

- pH optimum. The authors used HEPES buffer with a pH range of 3 to 11 to determine the pH optimum of RbmB activity. However, the HEPES buffer only has a pH range of 6.8-8.2. I recommend either deleting the corresponding Figure 3B or repeating the experiments using buffers with overlapping pH ranges. Sigma Aldrich provides a handy list of useful pH ranges of various buffers, see Buffer Reference Center (sigmaaldrich.com)

Thank you for the suggestion. We repeated the experiment using the Teorell and Stenhagen universal buffer which has a buffering range of 2-12 (doi:https://doi.org/10.1002/9783527659227.ch2) and updated the methods and references. The results were qualitatively very similar to our previous results.

Reviewer #3: 1. The authors describe RbmB as being specific. In particular, they write "This suggests that the specificity of the VPS/RbmB interaction is unlikely to be limited to Vc and may be shared with other Vibrio species that exhibit VPS-dependent biofilm formation (including Vibrio mimicus and Vibrio anguillarum)." However, I think this statement is stronger than has been proven. RbmB is tested against three other molecules, but is not tested against other bacterial EPS. If the action of RbmB is to specifically cleave VPS, it seems important to show that it does not cleave the EPS made by other bacteria. Conversely, it could be interesting if it acts specifically to EPS of many types. Note, I believe it may be possible to address this by better explaining the other molecules tested, and better qualifying the uncertainty of how specific RbmB truly is.

Thank you for raising this issue. We agree that without testing EPSs from other bacteria we can't say anything conclusive about RbmB's broad specificity. We did show in the paper that RbmB from *Vibrio coralliilyticus* is able to cut VPS from *Vibrio cholerae*, which is why we speculated that it might also cut the other Vibrio species. We modified the language to reflect the uncertainty. We modified our description of the other molecules tested (which differ from VPS), which shows that RbmB is not a generic glycosidase (at least against the limited number of polymers we tested). We modified the language in the passage that you quoted to remove the assertion that RbmB is specific. While we would love to test RbmB against other bacterial EPSs, there are questions about which and how many to use and it is very difficult to prove a negative (we do not expect it to cleave EPS molecules outside of VPS). So, we softened our assertion about RbmB specificity and left it as an unresolved question. We also referenced a new paper describing the gene organization of biofilm systems across different Vibrio species which shows that most of these systems contain RbmB-orthologs. We hope that this satisfies the reviewer's concern but are willing to engage on this question further if it does not.

2. The authors show that RbmB can disperse Vibrio cholerae biofilms when added exogenously in sufficient amounts. However, it is not shown that V. cholerae do this in vivo. The authors alude to this fact in the last paragraph of the discussion. However, much of the rest of the manuscript reads as if this is a demonstrated fact, e.g., the sentence "Finally, we confirmed that the cleavage strategy was used by Vc biofilms to disperse upon nutrient limitation." This seems too strong as the RbmB is added exogenously, rather than secreted as a "strategy" used by the biofilm to disperse. If the authors can show that WT biofilms disperse much faster than DeltaRbmB biofilms, then I think they could make a strong claim. I will again note that a clearer description of what was done and what is and is not known could be sufficient here.

We thank the reviewer for bringing up this excellent point. Indeed, how RbmB functions *in vivo* to facilitate dispersal is still a mystery and an active research area. It is still unclear whether it is secreted and degrades the VPS network (as it does in the exogeneous addition experiment) or functions intracellularly. What we do know is that RbmB is required for proper dispersal (see for example PMID: 17220218, PMID: 33288715). We have significantly updated the manuscript in relevant places.

We did show that WT biofilms disperse more readily than the Δ*rbmB* mutant biofilms. We updated Figure 6A to put the WT and Δ*rbmB* data side by side for direct comparison. Under the induction of dispersal by switching to M9 medium without a carbon source, WT biofilms lost 60% of its mass within 5 hours, whereas the Δ*rbmB* biofilm pretty much maintains the same biomass and therefore is defective in dispersal.

Part III – Minor Issues: Editorial and Data Presentation Modifications
Please use this section for editorial suggestions as well as relatively minor modifications of existing data that would enhance clarity.

Reviewer #1: • The results section entitled, "Recombinant-purified RbmB disrupts VPS/Bap1 aggregates," is confusing as written (particularly lines 172-178 describing the gel shift experiment with Bap1). Ultimately, the results in Figure 3 more convincingly demonstrate the enzymatic activity of RbmB against the VPS, compared to the two panels in Figure 2. I recommend that the authors consider either re-writing the description of the experimental approaches for Figure 2, namely how the Bap1 gel shifting experiment works, or simply remove these results from the manuscript as they are somewhat redundant with Figure 3 and are less compelling. Furthermore, the EM results in Figure 2 would be more convincing if a quantification of the monomers vs aggregates was performed.

We added more details to the results section to better explain how the gel-shift experiment worked. We agree that Figure 3 more conclusively characterizes the activity of RbmB against VPS, but argue that showing the effect in several different contexts (some qualitative and some quantitative) increases the rigor and reliability of this important finding. As asked by one reviewer (below), we did add quantification of Figure 1A.

In terms of the EM images, we find that the aggregation of VPS by Bap1 is a highly cooperative process that occurs over a narrow concentration range of Bap1. We have been unable to capture intermediates in this process. For this reason, the monomers vs. aggregates are all or nothing. We argue that the image (which is one of many) clearly shows monomers with Bap1 and with RbmB-treated VPS. With Bap1+untreated VPS we get aggregates and do not see free monomers.

• What is DsbA and how does it help with the purification of RbmB? Without this information the logic in the first paragraph is difficult to follow.

We added some a description detailing why we pursued using DsbA as a fusion partner. RbmB is predicted to contain two disulfide bonds and DsbA is a periplasmic protein that helps formation of disulfide bonds.

• Figure 5 is a difficult figure to interpret/read for non-specialists. It would be ideal to make it more legible (increase font size and the y-label in panel B in upside down). Can you point non-specialists to features of the plots that are relevant (as in Figure 4).

Thanks. We increased the font sizes of the axis labels and atom labels and rotated the Y-label. To help non-specialists, we added a green dotted line in the 2D spectrum to highlight the connectivity between carbon atoms on the gulose ring. The 2D data validate the assignments in the 1D data shown in the A panel. The main take-home message is that the C4, C5, and C6 carbon resonances shift largely downfield, which is consistent with the formation of a double-bond as described in the text.

• In Figure 6, the fluorescence images are blown-out/look of low quality. The authors should fix this. How was normalization performed in Figure 6B?

We have adjusted the contrast of the figure according to the reviewer's suggestion. Regarding the image quality, we have provided the high-res images in the full package. The original image may have lost some quality during conversion to pdf in the submission process.

There is no normalization performed in Figure 6B, as we are simply taking the ratio of biomass after and before nutrient limitation. This value can be in some cases slightly larger than unity because there could be some residual growth during the starvation period.

• In some areas of the manuscript language is rather informal for a final publication. A non-comprehensive list of specific examples include:

o Why not just type out V. cholerae and V. coralliilyticus, as is done for E. coli?

This was just a shortcut, but we have returned all mentions in the text to the longer version.

o Why do the authors repeatedly indicate milligram quantities of RbmB were purified (in the main text). This is fairly routine for recombinant protein purification and does not help the reader to interpret anything.

The author is a crystallographer and milligram quantities is a sort of codeword meaning enough for structural studies. Since this paper does not describe a crystal structure we removed mentions of milligram quantities.

o The term "mostly completing" on line 211 is ambiguous.

Understood. We replaced this with "reaching a plateau" and hope this is appropriate.

• Miscellaneous typos/word choice suggestions. This is a non-comprehensive list and I strongly suggest the authors carefully check for phrasing.

o In general, the authors could be more confident in describing established results about RbmB. For instance, on line 78, “suspected” should be changed to “known” as this is a well-established result.

Thank you for the suggestion; we have carefully checked for typos throughout the updated manuscript. We have replaced “suspected” to “known.”

o Line 77-78, “the V. cholerae gene cluster” should be “the V. cholerae biofilm gene cluster”.

Thanks – fixed.

o Line 112, “RbmB” should be “rbmB”. It is not possible to delete a protein.

Thank you for catching that. Fixed.

o Line 132 “uses” should be “use” and line 134, “results” should be “result”. In general, check that the correct verb tenses are used throughout the manuscript.

Thank you – fixed and checked.

o Line 212, remove “on”

Thanks for catching – fixed.

o Line 240, remove “obviously”

Removed.

Reviewer #2: - Figure 2A: Quantification of the Bap1/VPS complex abundance in each well would be helpful in understanding the native gel experiment.

Thank you for the suggestion. From our experience with this assay, we find a more linear response by monitoring the amount of the free Bap1 band than the Bap1/VPS band (the complex barely enters the gel and we might lose some material when the gel is disassembled for imaging). We included this quantification in the supplemental figures (figure S1).

- L206 and Figure S1: Please clarify which lines are +/- $CaCl_2$ in both the figure and figure legend.

Good catch – we fixed in the text and added a legend to the graph.

- L272: Explain what are Gal-Gul.

Thanks for pointing out this ambiguity. We spelled it out completely as galactose-gulose.

- Please change Figure S3 and S5 to Table S1 and S2.

We changed the labels and updated the text.

Reviewer #3: (No Response)
