# Supplementary material for: Vibrio cholerae RbmB is an α-1,4-polysaccharide lyase with biofilm-disrupting activity against Vibrio polysaccharide (VPS)
Source: PLoS Pathog. 2024 Dec 2;20(12):e1012750. doi: 10.1371/journal.ppat.1012750 (PMC11637428; doi:10.1371/journal.ppat.1012750)
Supplement: Attachment — Submitted filename: Responses_to_reviewers.docx [file ppat.1012750.s012.docx]

**We thank the reviewers for evaluating our revised manuscript. Based on the additional comments from Reviewer #2, we prepared a new manuscript which we hope satisfies the reviewer’s concerns. Please find responses to comments highlighted in blue.**

Reviewer's Responses to Questions

**Part I - Summary**
Please use this section to discuss strengths/weaknesses of study, novelty/significance, general execution and scholarship.

Reviewer #1: I am satisfied with the revisions made by the authors.

Thank you for evaluating our revised manuscript. We are glad that we were able to satisfy your concerns.

Reviewer #2: The revised manuscript by Weerasekera et al., “Vibrio cholerae RbmB is an α-1,4-polysaccharide lyase with biofilm-dispersal activity against Vibrio Polysaccharide (VPS)” focuses on the characterization of RbmB predicted to be a hydrolase and contribute to V. cholerae biofilm dispersion. Using a combination of biochemical and analytical tools, the authors demonstrated that RbmB acts as a polysaccharide lyase by cleaving VPS at a unique site into a mixture of tetramers and octamers. Tis reviewer enjoyed the enzymology and biochemical characterization of RbmB and the fate of Vps. The revisions have improved the clarity of the manuscript regarding the biochemical characterization of RbmB and the VPS analysis. And while most of the prior reviewers concerns were addressed, some concerns remain and/or the revised manuscript thanks to its improved clarity, raised a few additional questions. The main weakness for this reviewer is the contribution of RbmB to the dispersion of V. cholerae biofilms. Comments and concerns are discussed below.

Thank you for reading our revised manuscript carefully. We addressed your new concerns to the best of our ability. Please find our responses below. We realize that the word dispersion as it pertains to biofilms refers to a specific mechanism likely triggered through signaling and genetic means, and that while we might be seeing a dispersion-like phenotype, this may not be the same as *bone fide* dispersion. For this reason, in our manuscript we now refer to the RbmB-induced phenotype as “biofilm disruption.” We hope that the reviewer accepts this more generic term to describe the activity of recombinant RbmB on *V. cholerae* biofilms.

Reviewer #3: I thank the authors for their thorough responses to my questions, and those of the referees. I think this is a very interesting manuscript and strongly support its publication at this time.

Thank you for evaluating our revised manuscript.

**Part II – Major Issues: Key Experiments Required for Acceptance**
Please use this section to detail the key new experiments or modifications of existing experiments that should be absolutely required to validate study conclusions.

Generally, there should be no more than 3 such required experiments or major modifications for a "Major Revision" recommendation. If more than 3 experiments are necessary to validate the study conclusions, then you are encouraged to recommend "Reject".

Reviewer #1: None

Thanks!

Reviewer #2: 1. Previous reviewers commented on RbmB not having been shown to induce dispersion by V. cholerae biofilms in vivo and this reviewer found the response unsatisfactory. Lack of dispersion as seen for an ΔrbmB mutant does not mean that RbmB induces dispersion. There are many reasons why a biofilm may or may not disperse. And the findings reported here do not support RbmB inducing dispersion, but at most instead disassembling or disaggregation biofilms.

We understand your concern and while we expect that RbmB plays an important role in dispersal (as described in PMID: 33288715), we agree that we have not conclusively shown that the dispersal-like phenotype we see is true dispersal. For this reason, we changed the language in the paper to not describe this as dispersal, but rather as disruption.

2. The authors state “in several places that exogenously added recombinantly produced RbmB can efficiently disperse V. cholerae biofilms and that this findings provides direct support for the ability of RbmB to induce dispersion. See also title. However, this is not considered to be dispersion but disassembly or disaggregation, as it is not an active process, but passive due to assumed matrix degradation. This reviewer highly recommends replacing “disperse” with “disassemble” or “disaggregate”.

To address the reviewer’s concern, we have avoided the term dispersal to refer to experiments where we add exogenous RbmB. The exception is Figure 6A, which describes actual dispersal (as it has been described in PMID: 29056457 and PMID: 33288715**)**. We primarily use the term “digests” or “disrupts” rather than “disassemble” or “disaggregate”, because the latter two connote a possibly complex mechanism of disassembly. Since we have shown that the enzyme purely cleaves VPS into smaller fragments, we prefer to say digest or disrupt.

3. While the author demonstrate that exogenously added RbmB induces biofilm disaggregation, the authors indicated that this response was only observed upon depletion of nutrients. This finding raises several questions considering that nutrient depletion alone induce dispersion by the WT . 1) why does exposure to exogenously added RbmB not induce dispersion without the added dispersion cue of nutrient depletion? What is the appearance of rbmB mutant biofilms post 1 hour of nutrient depletion? What is the effect of heat inactivated RbmB in the absence and presence of nutrient depletion, following the same incubation time?

We previously performed all assays with nutrient depletion. The reason is to slow growth in these assays so colonies do not overgrow, thus complicating the assay. The appearance of rbmB mutant biofilms post 1 hour of nutrient depletion (with no added RbmB) is what is shown in the top right panel of Figure 6C – no significant change before or after nutrient limitation, indicating a defective dispersal phenotype. To address the reviewer’s concern regarding nutrient limitation, we repeated the assays without nutrient depletion (new Figure S6). Panel A shows that even without nutrient depletion, added recombinant RbmB disrupts Δ*rbmB* biofilms. As the reviewer suggested, we also tested heat inactivated RbmB (which we confirmed had no enzymatic activity using the reducing end quantification assay in Figure S6B) and saw no disruption activity.

4. An additional answered question is whether incubation of WT biofilms with recombinantly produced RbmB indeed disassembly biofilms by degrading VPS? This reviewer strongly suggests assessing the fate of VPS in vivo upon exposure to recombinantly produced RbmB.

Thank you for this suggestion. First, we showed in the updated manuscript that WT biofilms incubated with RbmB indeed are disrupted (new Figure S6C). To answer the other question, we stained biofilms with fluorescent wheat germ agglutinin (WGA), which is used widely to label VPS (PMID: 22798614) due to the GlcNAc unit in VPS (new Figure S7). We imaged biofilms with and without the addition of recombinant RbmB and see a dramatic decrease in the fluorescence signal of VPS that we visualize when RbmB is added. This result is consistent with RbmB digesting VPS within the same timeframe as biofilm disruption.

5. Similarly, the authors should consider treating pre-formed wild-type biofilms (not rbmB mutant) with different concentrations of purified RbmB and/or inactive RbmB and analyzing the biofilm disassembly rate. This might be a direct way of showing RbmB function, instead inducing dispersion using depletion of nutrient.

As suggested, we did this experiment – please see new Figure S6C. We grew WT biofilms without nutrient limitation and tested heat-inactivated RbmB and active RbmB at two different concentrations. We see a dose-dependent reduction in the amount of biofilm mass attached to the surface that is independent of nutrient limitation. This result demonstrates that the biofilm disruption that we see is caused by the activity of RbmB and not due to nutrient limitation.

6. Figure 6C - Previous reviewers commented on fluorescent images shown in Figure 6C to appear to be of different zoom which the authors assured was not the case. But what is the explanation of clusters shown in the top panel to appear larger than those in the panels below? The images represent biofilms formed by the same strain. Please explain.

The size of the biofilm clusters can be quite variable from day to day, from one biological replicate to the other – this is why we quantify the ratio of the total biofilm mass after and before RbmB treatment for statistical analyses. Just to be absolutely certain, we repeated the imaging experiment with three replicates and updated the data. Also, we updated the top panel in Figure 6C by showing the biological replicate in which the biofilm size is comparable to the other two panels.

7. For all graphs, please show results (data points) of individual repeats instead of just the average/stdev

We have now done this throughout the manuscript.

8. Figure legends and stats – indicate in the legend what was used as comparator for the statistical analysis and which statistical analysis was performed (e.g. Anova will be followed by t-tests for pair-wise comparisons with Tukey’s correction?

Some of this information was previously only in the methods. We have now added the detailed statistical information in the legend.

9. IT is unclear if experiments shown in Figure 6 were carried out using cleaved or uncleaved DsbA-RbmB. The information is neither provided in the Material section of the figure legend. Other experiments/methods provide this information.

This experiment was done with the uncleaved DsbA-RbmB. We see no difference in enzymatic activity whether DsbA is removed or not (see Figure S6B) – for convenience we mostly use the fusion protein. We made a note of this in the methods section to make this clearer. We have also confirmed that DsbA itself does not cleave VPS (Figure 6B) or disrupt biofilms (data not shown).

Reviewer #3: None.

Thanks!
